# Bicycle-related cervical spine injuries

**DOI:** 10.1016/j.xnsj.2022.100119

**Published:** 2022-04-30

**Authors:** Svend Filip Eng, Ingar Næss, Hege Linnerud, Pål Rønning, Tor Brommeland, Magnus Evjensvold, Terje Sundstrøm, Pål Galteland, Mats Døving, Mads Aarhus, Eirik Helseth, Jon Ramm-Pettersen

**Affiliations:** a Department of Neurosurgery, Oslo University Hospital, Oslo, Norway; b Institute of Clinical Medicine, Faculty of Medicine, University of Oslo, Oslo, Norway; c Department of Neuroradiology, Oslo University Hospital, Oslo, Norway; d Department of Neurosurgery, Haukeland University Hospital, Bergen, Norway; e Department of Clinical Medicine, University of Bergen, Bergen, Norway; f Department of Maxillofacial Surgery, Oslo University Hospital, Oslo, Norway

**Keywords:** Public health, Bicycling, Trauma, Surgery, Spine, Occipital condyle fracture, ASA-PS, American Society of Anesthesiologists physical status, CSI, Cervical spine injury, cSCI, Cervical spinal cord injury, OC-Fx, Occipital condyle fracture, Fx, Fracture, GCS, Glasgow coma scale, OFx, Odontoid fracture, OUH-U, Oslo University Hospital, Ullevål, TBI, Traumatic brain injury, Multiple trauma was defined as a simultaneous TBI (mild, moderate, or severe according to HISS) and/or imaging-proven (X-ray, CT, or ultrasound) injury in one or more of the following regions: face, thoracolumbar spine, chest, abdomen, pelvis or extremities. Skin injuries were not registered

## Abstract

**Background:**

Bicyclists are vulnerable road users. The aim of this paper was to describe all bicycle-related traumatic cervical spine injuries (CSIs) in the South-East region of Norway (2015–2019), and to investigate whether certain types of CSIs are typical for bicyclists.

**Methods:**

Retrospective cohort study of prospectively collected registry data of all CSIs in the South-East region of Norway (3.0 million inhabitants), from 2015 to 2019. Patient characteristics, injury types, and treatment were summarized with descriptive statistics. Bayesian multivariable logistic regression was used to identify potential factors associated with occipital condyle fractures (OC-Fx) or odontoid fractures (OFx).

**Results:**

During the five-year study period, 2,162 patients with CSIs were registered, and 261 (12%) were bicycle-related. The incidence of bicycle-related CSIs was 1.7/100,000 person-years. The median age of the patients with bicycle-related CSIs was 55 (IQR: 22) years, 83% were male, 71% used a helmet, 16% were influenced by ethanol, 12% had a concomitant cervical spinal cord injury (SCI), and 64% sustained multiple traumas. The three most common bicycle-related CSIs were C6/C7 fracture (Fx) (28%), occipital condyle Fx (OC-Fx) (23%) and C5/C6 Fx (19%). Patients with bicycle-related CSIs compared to patients with non-bicycle related CSIs were younger, more often male, had fewer comorbidities, more likely multiple traumas, more often had OC-Fx, and less often sustained an odontoid fracture (OFx). Multivariable logistic regression of potential risk factors for OC-Fx demonstrated a significantly increased risk of OC-Fx for bicyclists compared to non-bicyclists (OR=2.8).The primary treatment for bicycle-related CSIs was external immobilization in 187/261 (71.6%) cases, open surgical fixation in 44/261 (16.8%), and no treatment in 30/261 (11.5%).

**Conclusion:**

Bicycle crashes are a frequent cause of CSIs in the Norwegian population and should be of concern to the public society. The three most common bicycle-related CSIs were C6/C7 fracture, occipital condyle fracture and C5/C6 fracture.

## Highlights

• The incidence of bicycle-related cervical spine injuries (CSI) was 1.7/100,000/year.
• Bicycling was the second most common cause of CSI, only preceded by falls.
• Occipital condyle fracture was common in bicyclists.
• Bicyclists with CSI were associated with more multiple trauma and concomitant head injury than non-bicyclists.

## Introduction

Bicycling is a popular leisure activity and an effective mode of transport for short- and medium-range travel [1, 2]. Bicycling is associated with individual health benefits and improved intercity transportation logistics, and has important environmental benefits due to less congestion [3, 4]. However, bicyclists are vulnerable road users, and the number of bicycle-related injuries has increased over the last decade [5], [6], [7]. Most bicycle injuries are reported as either minimal or mild with little or no permanent late effects [8, 9]. Nevertheless, a steady rise in the number of more severe bicycle injuries has been observed [5]. Bicycle-related traumatic brain injuries (TBIs) and spinal injuries entail a higher risk of death and permanent late effects versus injuries to other parts of the body [10], [11], [12].

The incidence of traumatic cervical spine injury (CSI) in the Norwegian population is 14.4/100,000 person-years, and 12% of these injuries are bicycle-related. Thus, bicycling is now the second most common cause of CSI, preceded only by falls [13], [14], [15]. Functionally, the cervical spine extends from the craniocervical junction (occipital condyle (OC)/atlas (C1) to the cervicothoracic junction (C7/Th1)). The most feared complication of CSI is spinal cord injury (SCI), which is associated with increased mortality and permanent morbidity [16]. The treatment of CSI depends on the morphology of the fracture, discoligamentous status, associated neurological injury (SCI and/or cervical radiculopathy), and the patient's comorbidity load [17], [18], [19], [20], [21]. The treatment options are either external immobilization or surgical fixation, although in some cases, no stabilization is needed.

In this population-based study, we present an overview of all bicycle-related CSIs in a defined Norwegian population of 3 million people for the time period from 2015–2019. To reveal special characteristics for bicycle-related CSIs, we compared bicycle-related CSIs with all non-bicycle CSIs in the same time period.

## Materials and methods

Oslo University Hospital-Ullevål (OUH-U) is a level 1 trauma center in Oslo, Norway and serves as the central trauma-care facility for the South-East region of Norway, which cover 3 million people. OUH-U performs all surgeries for cervical spine injury (CSI) in this region. There are 20 hospitals in the region with general and/or orthopedic surgeons and radiological services that refer patients with CSIs to OUH-U. The included patients were either admitted to OUH-U for treatment, or non-surgical treatment was carried out locally after consultation with the Department of Neurosurgery at OUH-U.

Since January 1, 2015, the Department of Neurosurgery at OUH-U has maintained a prospective population-based quality control registry including all CSIs, from the occipital condyle (C0)/C1 to C7/Th1 [15]. Cervical spine injury (CSI) is defined as any traumatic fracture in the cervical vertebras or occipital condyles diagnosed with cervical-CT. In addition, we have included cases with MRI verified traumatic discoligamentous injury (and no fracture) if they were associated with neurological injury (spinal cord injury or radiculopathy), or were in need of stabilization. Only patients with an 11-digit unique Norwegian Social Security Number who were registered as residents within the area of the South-East region of Norway were included. The completeness of the CSI registry has previously been evaluated by comparing the registry data with data from the Norwegian Patient Registry [13]. The completeness was 100% for CSIs in need of surgical fixation and >90% for CSIs treated with external immobilization or no treatment. In sum, nearly all CSIs are included in the registry, and there is most likely no selection bias of importance.

From January 1, 2015 to December 31, 2019, 2,162 patients with CSIs were registered. The following data were extracted: date of injury, sex, age at time of injury, pre-injury American Society of Anesthesiologists (ASA) score [22, 23], injury mechanism (bicycle-related versus non-bicycle related), type of bicycle (bicycle, electric bicycle, electric scooter), alcohol influence at the time of injury (yes/no/unknown), type/level of CSI (occipital condyle (C0), C1, C2-odontoid, C2-Hangman, C2-other, C2/C3, C3/C4, C4/C5, C5/C6, C6/C7, C7/Th1), cervical spinal cord injury (cSCI) (no/incomplete/complete), cSCI graded according to the American Spinal Injury Association (ASIA) Impairment scale (AIS) (from A – no neurologic function to E – full neurological recovery) [24], traumatic brain injury (TBI) classified according to the head injury severity scale (HISS) [25], multiple traumas (yes/no/unknown), primary treatment (external immobilization with stiff collar alone, open surgical fixation, or no treatment).

Multiple trauma was defined as a simultaneous TBI (mild, moderate, or severe according to HISS) and/or imaging-proven (X-ray, CT, or ultrasound) injury in one or more of the following regions: face, thoracolumbar spine, chest, abdomen, pelvis or extremities. Skin injuries were not registered.

This quality control study was approved by the Data Protection Officer (DPO) at Oslo University Hospital and patient consent was waived (DPO approval #19/20770). This study is exempt from an application to the Regional Ethical Committee. Data were extracted from our hospital-approved quality database for CSIs (DPO approval #2014/12304). The database is kept in Medinsight and maintained by the Department of Neurosurgery at OUH-U.

Patient characteristics, injury types, and treatment are summarized with descriptive statistics. Categorical data are presented as frequencies and percentages. Continuous variables are presented using the mean or median, depending on the distribution. To compare group differences, we employed the Pearson χ2 test for categorical variables and the independent t-test or the Mann–Whitney U-test for continuous variables.

Bayesian multivariable logistic regression was used to identify potential factors associated with OC-Fx or Ofx. Weakly informative priors -N (0, 2.5) with automatic rescaling (depending on the distribution of the variable) were used in the analysis for both intercept and coefficients. The model coefficients were visualized as forest plots. The mild, moderate, and severe head injury severity categories are referenced against none/minimal head injury (HISS scale). Males are referenced against females, ASA 3-5 against ASA 1-2, and bicycle versus non-bicycle mechanisms of injury. To further improve legibility, we computed the marginal effect plots displaying the effect of age and head injury severity on the different fractures.

IBM SPSS statistics, version 25 (IBM Corp., Armonk, NY), R v3.6, and STATA SE were used for all analyses. P-value <0.05 were considered to be significant.

## Results

**Fig. 1:**
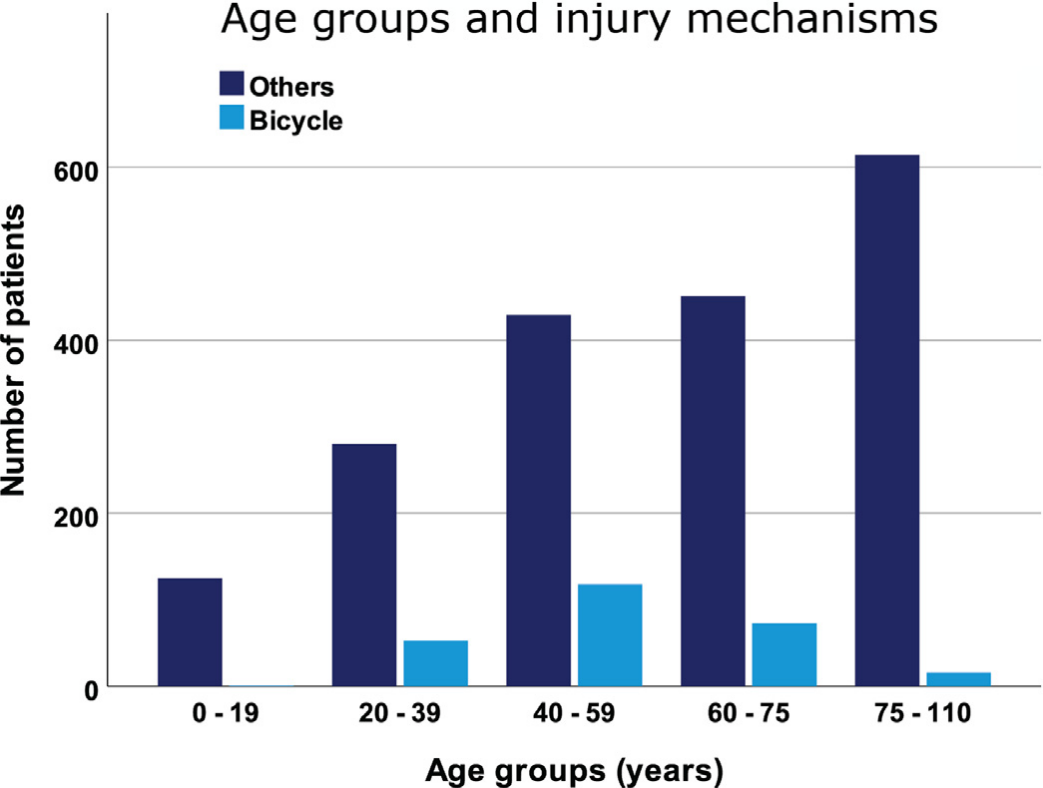
All CSIs in the South-East region of Norway for 2015 - 2019 according to age group and injury mechanism (bicyclists versus non-bicyclists). N = 2162.

In our defined population of 3 million people (South-East region of Norway), we prospectively registered 2,162 patients with CSIs during the 5-year period from 2015–2019. Of these CSIs, 261 (12%) were bicycle-related. The total incidence of CSI was 14.4/100,000 person-years, and the incidence of bicycle-related CSIs was 1.7/100,000 person-years. Compared to non-bicycle CSI patients, bicycle CSI patients were younger, more often male, had fewer comorbidities (preinjury ASA) and more often suffered from multiple traumas (Table 1, Fig. 1).

**Table 1:** Patient characteristics for bicyclists and non-bicyclists with cervical spine injuries (CSI) in the South-East region of Norway during the time period 2015 – 2019.

		AllN (%) 2162 (100)	Non-bicycle CSIN (%)1901 (100)	Bicycle CSIN (%)261 (100)	Statistics
**Sex**	Male	1461 (67.6)	1244 (65.4)	217 (83.1)	P<0.001
	Female	701 (32.4)	657 (34.6)	44 (16.9)	
**Mean age**	Years	59.1 years	59.9 years	53.3 years	P<0.001
**Pre-injury ASA**^1^	1-2	1278 (59.1)	1054 (55.4)	224 (85.8)	P<0.001
	3-5	783 (36.2)	759 (40.0)	21 (8.1)	
	Unknown	101 (4.7)	88 (4.6)	16 (6.1)	
**Cervical injury**	OC-Fx^2^	219 (10.1)	158 (8.3)	61 (23.4)	P<0.001
	C1- Fx	246 (11.4)	217 (11.4)	29 (11.1)	NS
	OFx^3^	419 (19.4)	403 (21.2)	16 (6.1)	P<0.001
	C2 – Hangman Fx	60 (2.8)	56 (2.9)	4 (1.5)	NS
	C2 – Other Fx	141 (6.5)	127 (6.9)	14 (5.4)	NS
	C2/C3 injury^4^	46 (2.1)	41 (2.2)	5 (1.9)	NS
	C3/C4 injury	175 (8.1)	145 (7.6)	30 (11.5)	NS
	C4/C5 injury	251 (11.6)	215 (11.3)	36 (13.8)	NS
	C5/C6 injury	368 (17.0)	318 (16.7)	50 (19.2)	NS
	C6/C7 injury	571 (36.3)	497 (26.1)	74 (28.4)	NS
	C7/Th1injury	178 (8.2)	153 (8.0)	25 (9.6)	NS
**cSCI**	Yes	250 (11.6)	219 (11.5)	31 (11.9)	NS
**Level of cSCI**	OC-C2	21 (1.0)	20 (1.1)	1 (0.4)	NS
	C3-C7	229 (10.6)	199 (10.5)	30 (11.5)	NS
**Multiple trauma**	No	951 (44.0)	870 (45.8)	81 (31.0)	P<0.001
	Yes	1076 (49.8)	908 (47.8)	168 (64.4)	
	Unknown	135 (6.2)	123 (6.5)	12 (4.6)	

1 Pre-Injury ASA – Pre-Injury American Society of Anesthesiologists Physical Status Classification

2 OC-Fx – Occipital condyle fracture

3 OFx – Odontoid fracture

4 Injury – Fx and/or discoligamentous injury with potential instability at affected level, e.g. C2/C3, C3/C4 etc.

Our main focus was 261 patients with bicycle-related CSIs; 253 had bicycle injuries, 8 had electric bicycle injuries, and 0 had electric scooter injuries. The median age was 55 (IQR: 22, range: 16-87), and 83% were male. The number of bicycle-related CSIs peaked in the 40-59 age group, while few CSIs were observed in bicyclists <20 or ≥75 (Fig. 1). Men were overrepresented in all age groups (Table 1).

Concomitant cSCI was registered for 31/261 (11.9%) bicyclists, being complete in 4/31 (13%) and incomplete in 27/31 (87%) of these bicyclists. Most cSCI was secondary to subaxial cervical injuries in both the bicyclists and non-bicyclists (30/31 and 199/229 (p=0.111)) (Table 1). The severity of the cSCI, graded according to the AIS, is provided in Table 2.

**Table 2:** Grading of cSCI^1^ in bicyclists and non-bicyclists with CSI^2^ according to AIS^3^.

AIS	AllN (%) 2162 (100)	Non-bicycle CSIN (%)1901 (100)	Bicycle CSIN (%)261 (100)	Statistics
**A**	36 (1.7)	32 (1.7)	4 (1.5)	NS
**B**	35 (1.6)	31 (1.6)	4 (1.5)	
**C**	72 (3.3)	60 (3.2)	12 (4.6)	
**D**	107 (4.9)	96 (5.0)	11 (4.2)	
**E**	1911 (88.4)	1681 (88.4)	230 (88)	
**Missing**	1 (0.05)	1 (0.05)	0 (0.00)	

1 cSCI – cervical spinal cord injury

2 CSI – cervical spine injury

3 AIS – American Spinal Injury Association (ASIA) Impairment Scale

Multiple traumas with CSIs were seen in 168/261 (64.4%) bicyclists (Table 1). The four most frequently associated injuries were TBI (48%), facial Fx (26%), thoracolumbar Fx (22%), and thoracic injury (18%) (Table 3). Information on ethanol influence at the time of injury was available for 231/261 (89%) bicyclists, among whom 38/231 (16%) were under the influence.

**Table 3:** Multiple traumas. Overview of concomitant injuries in bicyclist with CSI^1^ (unknown multiple trauma status in 12/261).

Injury region	N (%)249 (100%)
TBI^2^	120 (48.2)
Facial fracture	64 (25.7)
Thoracolumbar fracture	55 (22.1)
Thoracic injury^3^	45 (18.1)
Extremity fracture	33 (13.3)
Abdominal injury^4^	5 (2.0)
Pelvic fracture	4 (1.6)

1 CSI – Cervical spine injury

2 TBI – Traumatic brain injury (Mild, moderate or severe)

3 Fracture, pneumothorax, hematothorax, and/or lung contusion

4 Image or surgery verified injury of abdominal content

**Fig. 2:**
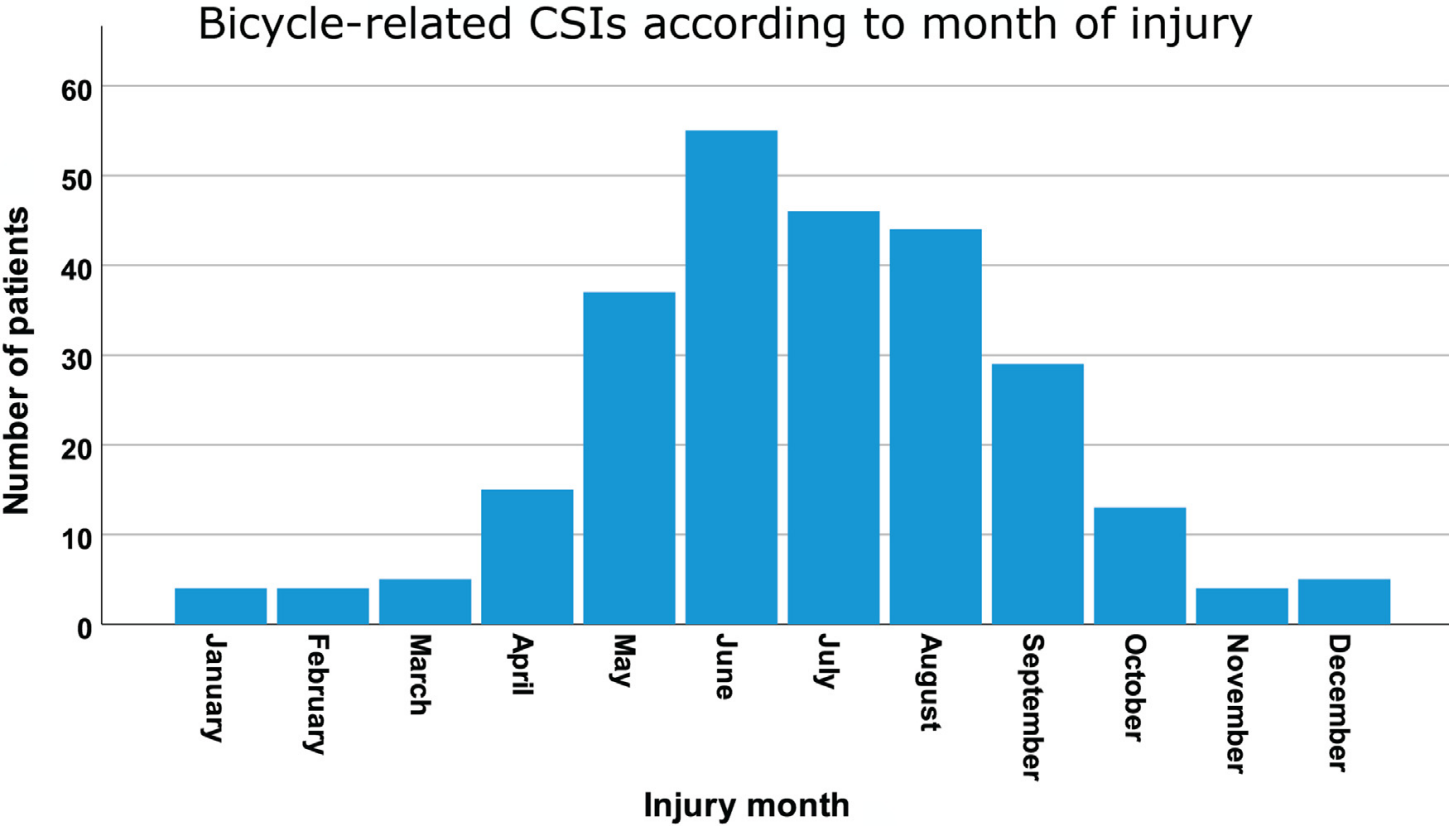
Bicycle-related CSIs according to month of injury. N = 261.

The bicycle-related CSIs showed typical seasonal variations, reflecting the climate in Norway with low temperatures and snow/ice in the winter season, lasting from November to March. The rate increased during spring, peaked in summer, fell in autumn, and showed a stable low rate during winter (Fig. 2B).

Table 1 contains a description of the bicycle-associated CSIs and a comparison between the bicycle group and the non-bicycle group. The most common CSIs for bicyclists were C6/C7 Fx, followed by occipital condyle fracture (OC-Fx) and C5/C6 Fx. When we compared the types of CSIs among the bicyclists and non-bicyclists, it seemed that bicycle injuries were associated with an increased risk of OC-Fx and a reduced risk of OFx (Table 1). In bicyclists with OC-Fx, 60/61 were unilateral and 1/61 bilateral. Of the unilateral OC-Fx, 31 were on the right side and 29 on the left side (p=0.80). No bicyclists presented with occipito-cervical dissociation.

**Fig. 3:**
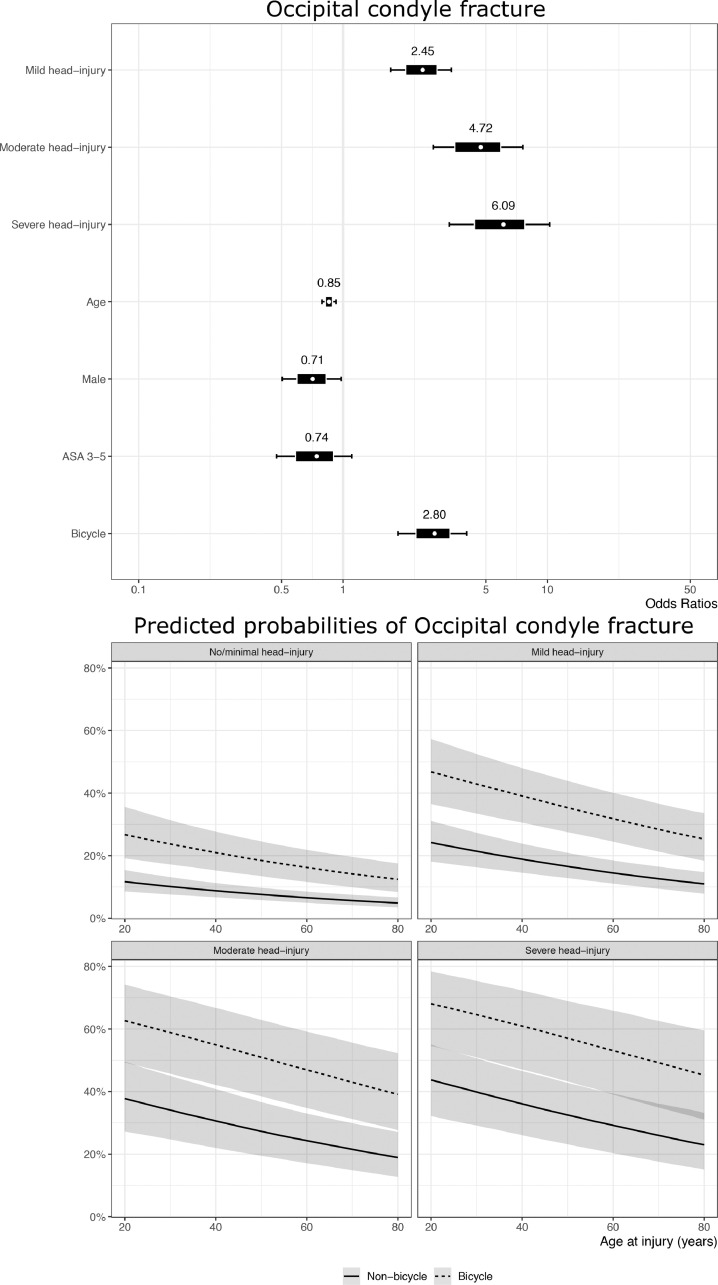

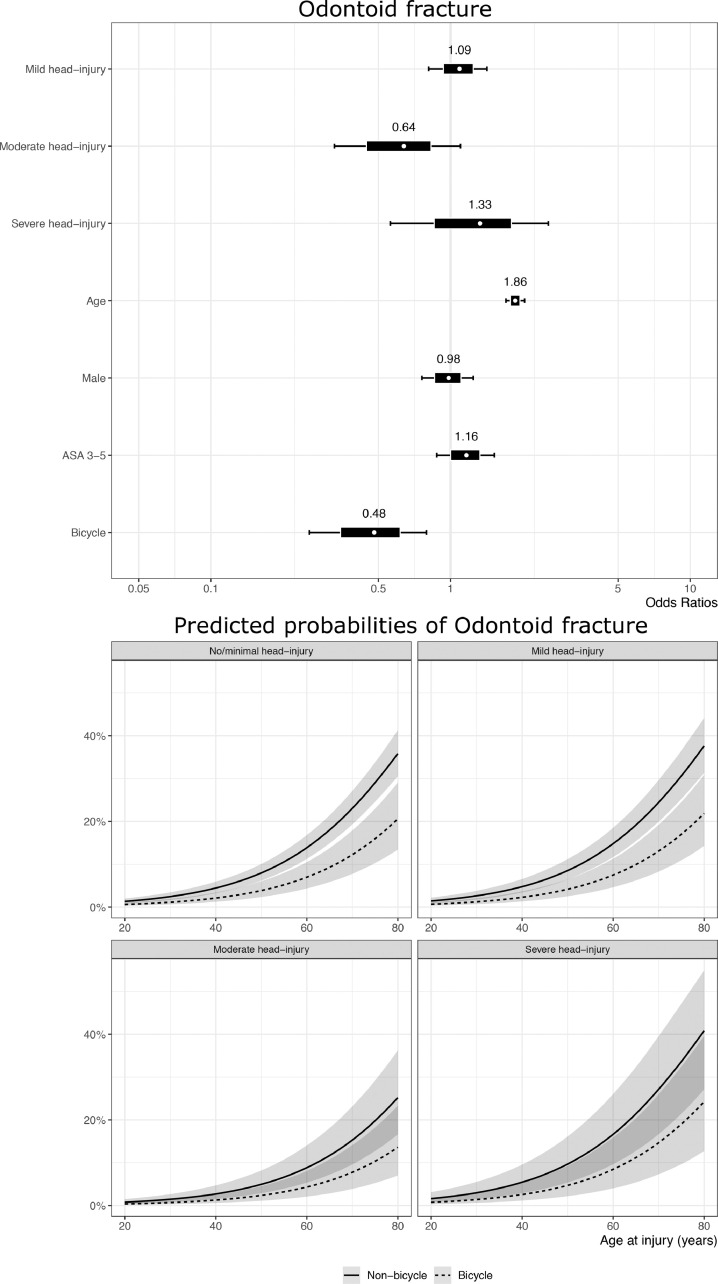
Multivariable logistic regression analysis to evaluate the potential association between the injury mechanism (bicyclists versus non-bicyclists) and the risk of either OC-Fx (A) or OFx (B). The forest plots (the upper part of figures) visualize the coefficients obtained from the Bayesian multivariable logistic regression models. The marginal effect plots (lower part of the figures) of increasing age on the probability of fracture stratified via the HISS demonstrate the different effects of age and HISS score on fracture probability for OC-Fx and OFx. Bicyclists had a significantly increased risk of OC-Fx compared to non-bicyclists, while older age was the main factor associated with an increased risk of OFx.

To better evaluate the potential association between the injury mechanism (bicycle versus non-bicycle) and the risk of either OC-Fx or OFx, we performed a multivariable logistic regression analysis including age, sex, comorbidity, severity of head injury, and injury mechanism (Fig. 3A-B). The multivariable analysis of potential risk factors for OC-Fx among all CSI patients (bicycle-related and non-bicycle related) demonstrated a significantly increased risk of OC-Fx for bicyclists compared to non-bicyclists (OR=2.8, 95% credible interval (CI) [1.9, 4.0]. OC-Fx was also associated with concomitant increasing head injury severity (OR=2.5, 95% CI [1.7, 3.4] for mild TBI, OR=4.7, 95% CI [2.8, 7.6] for moderate TBI, and OR=6.1, 95% CI [3.3, 10.3] for severe TBI, in reference to non- or minimal head injury). A similar multivariable logistic regression analysis of potential risk factors for OFx indicated that the older age was the main factor associated with an increased risk of OFx (Fig. 3B).

Information regarding helmet use was available for 185/261 (71%) bicyclists, of whom 131 (71%) used helmets and 54 (29%) did not. Neither sex nor age was significantly associated with helmet use, but ethanol-influenced bicyclists had a significantly lower rate of helmet use (20% versus 80%) (p=0.001). Helmet use displayed no association with OC-Fx (p=0.806), OFx (p=0.275), or concomitant cSCI (p=0.371), but helmet users had a lower rate of concomitant TBI than non-helmeted bicyclists (62% versus 85%) (p=0.029).

The primary treatment of bicycle-related CSIs was external immobilization with a stiff collar alone in 187/261 (71.6%) injured bicyclists, open surgical fixation in 44/261 (16.8%), and no treatment in 30/261 (11.5%). The majority of patients in the “no treatment group” had an isolated fracture of a spinous or transverse process. Six patients in the external immobilization group later underwent open surgical fixation.

## Discussion

The incidence of bicycle-related CSIs in the general Norwegian population was 1.7/100,000 person-years. Compared to patients with non-bicycle related CSIs, bicyclists were younger, more often male, had fewer comorbidities, and more often suffered from multiple traumas. The number of bicycle-related CSIs peaked in the 40-59 age group, while we noted very few bicycle-related CSIs among those <20 and ≥75. OC-Fx was more common among the cyclists than in the non-bicyclist group. These fractures were also associated with increasing severity of concomitant TBI. Concomitant cSCI was present in 12% of the bicyclists. A helmet was worn by 71% of the bicyclists, although less often among those who had consumed alcohol. Wearing a helmet was associated with a lower rate of concomitant TBI.

**Incidence and seasonal variations:** We did not identify any previous studies reporting on the incidence of bicycle-related CSIs in the general population. We would expect the rate of CSIs to depend on several factors such as the share of inhabitants riding bicycles, climate, population density, population age, culture, the social economy, and political efforts to stimulate bicycling and improve road safety.

Countries such as Denmark and the Netherlands have a greater proportion of inhabitants who use bicycles for their daily commute than Norway [26, 27]. Nevertheless, the number of inhabitants in Norway who ride bicycles seems to be on the rise, particularly in urban areas [28]. The increasing numbers of bicyclists, especially in rush-hour traffic, have resulted in more patients with bicycle-related injuries [5]. Bicyclists have a higher risk of injury per kilometer traveled than car occupants [29]. In our total CSI patient population, bicycle crashes were the second most common cause of CSI preceded only by falls [15]. In the US, bicycle-related injuries account for 81% of all adult sports-related spinal injuries [30].

**Gender and age:** Among patients with bicycle-associated CSIs, there was a large overrepresentation of men across all age groups, peaking in the 40-59 age group. The main reason for male overrepresentation is most likely a combination of a higher share of males using bicycles on an everyday basis than females, and a sex difference in everyday risk-taking behavior [8, 31].

Notably, only one of the 261 bicyclists with CSIs was younger than 20, despite the frequent use of bicycles as a mode of transport and recreation among children and adolescents [5]. Hence, it seems that the risk of sustaining a CSI is lower for children and adolescents than adults. Children and adolescents have less calcified skeletons and a stronger periosteum than adults, resulting in a more elastic and less brittle skeleton. In addition, children cycle at lower speeds and weigh less than adults; as such, children are likely to experience less force during a crash. These factors combined might explain the low incidence of bicycle-related CSIs in children and adolescents.

**Morphology of bicycle-associated CSIs:** OC-Fx appeared to be a typical cyclist fracture. We believe the mechanism behind the OC-Fx in bicyclists is a combination of rotation and compression forces in the C0/C1 joint as the cyclist goes over the handlebars and hits the ground head first [32]. The association between the severity of head injury and the risk of OC-Fx lends support to this theory. OFx is a fracture type commonly seen in elderly, comorbid patients with osteoporosis [19]. This is a group of people who seldom utilize a bicycle, and we believe this explains why OFx is rare among bicyclists. Concomitant cSCI was present in 12% of bicycle CSIs, similar to non-bicycle injuries.

**Helmet:** A large proportion of Norwegian bicyclists wear helmets even if not obliged by law [5, 8, 33]. However, we noted that bicyclists who had consumed alcohol were less inclined to wear helmets, which is in line with previous findings [34]. Helmeted bicyclists with CSIs had a significantly lower rate of concomitant TBI than non-helmeted bicyclists. This finding corresponds with the established role of helmets in preventing TBI [35], [36], [37], [38], [39].

Recent data indicate that wearing a helmet also reduces the risk of midface fractures, but not mandibular fractures or dentoalveolar injuries [40, 41]. The role of helmet use in the prevention of CSI is less clear. The literature reports divergent results, from an increased risk of CSI with helmet use, via no protective effect of the helmet, to a decreased risk of CSI when wearing a helmet [35, 38, [42], [43], [44], [45], [46]]. We did not design our study to detect whether helmets protect bicyclists against CSIs.

The use of bicycle helmets with a multidirectional impact protection system (MIPS) is increasing, but at a rather slow pace due to price. Thus, it is too early to conclude whether this type of helmet protects against TBI and CSI in vivo [47]. Another interesting protection device is the combined helmet/neck brace airbag (e.g., the Swedish Hövding 3) [48]. To date, the use of this airbag system is so limited that it is too early to decide if this will become a real alternative to more conventional helmets.

**Alcohol use:** Ethanol influence at the time of injury was registered in 16% of bicyclists with CSIs. This is somewhat lower than the rate of ethanol influence reported for patients with TBI regardless of the injury mechanism [49, 50]. Increased public awareness of the dangers associated with bicycling and alcohol consumption is nevertheless warranted.

**Electric bicycles and electric scooters:** In recent years, electric bicycles (legal in Norway with a maximum speed of 25 km/h) and electric scooters (legal in Norway with a maximum speed of 20 km/h), both classified as bicycles have become very popular. The sale of electric bicycles has risen substantially in the last five years [51]. According to our results, very few CSIs were associated with the use of electric bicycles. Nevertheless, due to the increasing share of electric bicycles—which easily reach higher speeds than conventional bicycles—a rising trend in electric bicycle-related CSIs may be expected in the years to come [52]. The use of electric scooters has risen immensely since 2018, especially in urban areas. The Oslo Accident and Emergency Outpatient Clinic's Section for Orthopedic Emergency reported in 2019 an alarming increase in injuries due to electric scooters following legislation of public scooter sharing systems in Oslo [53]. As of December 31, 2019, there were no registered CSIs related to an electric scooter crash in our database.

**Implications:** The government aims to increase commuting by bicycles due to the associated health benefits, potential reduction in rush hour traffic jams, and environmental advantages [1]. However, the increasing number of patients with serious bicycle injuries, including TBI and CSI, should be of concern to public society and politicians alike [5, 11]. Hence, road safety for bicyclists should receive greater attention to reach the political goal of attracting more people to use bicycles for their work commutes [27].

Improved road safety for bicyclists may be achieved through enhanced infrastructure, more bicyclists wearing helmets, zero tolerance for alcohol consumption prior to bicycling, and further development of protective equipment for bicyclists [54], [55], [56], [57]. Bicycles with defective brakes or bicycling in the dark without a light and/or reflective clothing are other issues that should be addressed [58].

**Strengths and limitations:** One strength of this study was the use of a prospective population-based database. However, the database was not designed specifically for this study. Variables describing type of bicycling, speed at the time of the crash, bicycling experience, light conditions, the bodyweight of the bicyclist, or type of crash were not available in the database, and this represents a limitation of the study. The generalizability of the findings might be limited to countries with comparable bicycle habits and road safety measures.

## Conclusions

Bicycle crashes are a frequent cause of CSIs in the Norwegian population and should be of concern to public society and politicians alike. The three most common bicycle-related CSIs were C6/C7 fracture, occipital condyle fracture and C5/C6 fracture. Bicycle injuries compared to non-bicycle injuries are associated with an increased risk of occipital condyle fracture.

## Funding statement

Ingar Næss received a scholarship from The Medical Student Research Program through the Medical Faculty, University of Oslo. The funding body has not influenced any aspects of the study design, data gathering or interpretation of this paper.

## Data availability

The research data are confidential because they contain sensitive information about our patients, which can lead to the identification of individual patients. According to regulations, the data cannot be made publicly available. However, the data can be made available from the authors upon reasonable request and with permission of the Data Protection Officer at OUH.

## Declaration of Competing Interest

The authors declare that they have no known competing financial interests or personal relationships that could have appeared to influence the work reported in this paper.

## Appendix Supplementary materials

Image, application 1

Image, application 1

Image, application 1

Image, application 1

Image, application 1

Image, application 1

Image, application 1

Image, application 1

Image, application 1

Image, application 1

Image, application 1

Image, application 1

Image, application 1
