# Supplementary material for: Bicycle-related cervical spine injuries
Source: N Am Spine Soc J. 2022 Apr 30;10:100119. doi: 10.1016/j.xnsj.2022.100119 (PMC9108519; doi:10.1016/j.xnsj.2022.100119)
Supplement: Supplementary file 1 [file mmc1.pdf]

ICMJE Form for Disclosure of Potential Conflicts of Interest

Instructions

In accordance with NASS' Universal Disclosure Policy, *The Spine Journal* expects all authors complete the following process to ensure that every manuscript submitted to *The Spine Journal* includes the necessary Conflict of Interest disclosure. The form is designed to be completed and stored electronically and contains four parts.

Note: Corresponding authors are responsible for ensuring each author receives the link to this document and that they provide a completed form. They are also responsible for uploading all author disclosures. Submissions with missing or outdated disclosures ***will not*** *be reviewed.*

1. Identifying Information

Enter your full name. If you are NOT the corresponding author please check the box "no" and enter the name of the corresponding author in the space that appears. Provide the requested manuscript information. Double-check the manuscript number and enter it.

2. Relationships Pertaining to Submitted Manuscript

This section asks for information about the work you are submitting for publication. **The time frame for this reporting is that of the work itself, from the initial conception and planning to the present.** The requested information is about resources that you received, either directly or indirectly (via your institution), to enable you to complete the work. Checking "No" means that you did the work without receiving any financial support from any third party -- that is, the work was supported by funds from the same institution that pays your salary and that institution did not receive third-party funds with which to pay you. If you or your institution received funds from a third party to support the work, such as a government granting agency, charitable foundation or commercial sponsor, check "Yes". Then complete the appropriate boxes to indicate the type of support and whether the payment went to you, or to your institution, or both.

3. Financial Relationship (Universal Disclosure)

The NASS disclosure policy requires authors to disclose estimated dollar amounts **to the nearest $1000 for activities taking place in the previous 36 months.** Financial disclosures in the *The Spine Journal* will be shown in ranges of estimated dollar amounts. NASS will translate estimated amounts submitted by authors into ranges for presentation in *The Spine Journal*. NASS respects that there are confidentiality agreements, legal issues, and practical barriers that might prevent public disclosure of more detailed information. The disclosure format, however, retains the intent of the policy: to disclose any and all participant relationships and to allow readers to judge whether or not a relationship constitutes a conflict of interest or source of bias with respect to information that is presented in the manuscript. *The Spine Journal* authors are required to disclose all relationships with industry, including relationships in negotiation. Relationships still in the negotiation phase will be classified with the same terminology on the basis of estimated potential future value. For further guidance and explanation regarding this policy and ramifications of policy violations, please refer to the the NASS Disclosure Policy (revised June 2011).

4. Other Relationships

Use this section to report other relationships or activities that readers could perceive to have influenced, or that give the appearance of potentially influencing, what you wrote in the submitted work.

ICMJE Form for Disclosure of Potential Conflicts of Interest

Section 1. Identifying Information

1. Given Name (First Name)
Eirik

2. Surname (Last Name)
Helseth

3. Date (use drop-down menu)
11-May-2021

4. Are you the corresponding author? ☐ Yes ☑ No

Corresponding Author's Name
Ingar Næss

5. Manuscript Title
Bicycle-related cervical spine injuries: Occipital condyle fractures are common in cyclists

6. Manuscript Identifying Number (if you know it)

Section 2. Relationships Pertaining to the Submitted Manuscript

Did you or your institution at any time receive payment or services from a third party for any aspect of the submitted work (including but not limited to grants, data monitoring board, study design, manuscript preparation, statistical analysis, etc…)?

Complete each row by checking "No" or providing the requested information. If you have more than one relationship click the "Add" button to add a row. Excess rows can be removed by clicking the "X" button.

Relationships Pertaining to Submitted Manuscript

Type	No	Money Paid to You	Money to Your Institution*	Name of Entity	Dollar Amount**	

* This means money that your institution received for your efforts on this study.
** Dollar amount, number of shares, and/or percentage of ownership.

Section 3. Financial Relationships (Universal Disclosure)

Place a check in the appropriate boxes in the table to indicate whether you have financial relationships (regardless of amount of compensation) with entities as described in the instructions. Use one line for each entity; add as many lines as you need by clicking the "Add +" box. You should report relationships that were present during the 36 months prior to submission.

Complete each row by checking "No" or providing the requested information. If you have more than one relationship click the "Add" button to add a row. Excess rows can be removed by clicking the "X" button.

Relevant financial activities outside the submitted work

ICMJE INTERNATIONAL COMMITTEE of MEDICAL JOURNAL EDITORS

ICMJE Form for Disclosure of Potential Conflicts of Interest

Relevant financial activities outside the submitted work						
Type of Relationship (in alphabetical order)	No	Money Paid to You	Money to Your Institution*	Entity	Dollar Amount**	
Type of Relationship (in alphabetical order)	No	Money Paid to You	Money to Your Institution*	Entity	Dollar Amount**	

* This means money that your institution received for your efforts.
** Dollar amount, number of shares and/or percentage of ownership.
^ Indicate whether amount received was towards investigator salary and/or staff/materials.

Section 4. Other relationships

Authors should disclose other relationships, which would reasonably be judged to have a direct relationship to the topic of the activity. These relationships that could reasonably be judged by an observer to be related to the topic should be disclosed through general disclosure (ie, estimated dollar amounts are encouraged but not required).

☑ No other relationships/conditions/circumstances that present a potential conflict of interest

☐ Yes, the following relationships/conditions/circumstances are present (explain below):

At the time of manuscript acceptance, journals may ask authors to confirm and, if necessary, update their disclosure statements. On occasion, journals may ask authors to disclose further information about reported relationships.

Show All Table Rows SAVE

Evaluation and Feedback

Please visit http://www.icmje.org/cgi-bin/feedback to provide feedback on your experience with completing this form.
